# Influence of Drugs Given to Nurses or Mothers on Their Suckling Infants

**Published:** 1887-02

**Authors:** 


### *INFLUENCE OF DRUGS GIVEN TO NURSES OR MOTHERS ON THEIR SUCKLING INFANTS.*

We abstract from *Les Nouveaux Remèdes* of August 1, 1886, the following interesting discussion of Dr. Fehling relating to the influence of certain drugs given to nurses on their suckling babies.

1. *Salicylate of Sodium.*—Dose varying between thirty and forty-five grains. Whenever the child is put to the breast one hour or less after the administration of the drug, the salicylate of sodium can be found in the child's urine. After the expiration of twenty-four hours, no traces of it can be found in the urine. Likewise, the salt cannot be recovered if the child is put to the breast very soon after the exhibition of the drug. The elimination of the drug terminates simultaneously in nurse and child.

2. *Iodide of Potassium.*—The same results are obtainable. The milk, if analyzed, gives the characteristic re-action. In the child, the elimination lasts seventy-two hours; in the nurse, forty-four hours.

3. *Ferrocyanide of Potassium.*—The re-action is very distinct in the urine of the nurse, but wholly absent in the child's urine.

4. *Iodoform.*—After prolonged application of iodoform upon wounds of the vagina or vulva, iodine can be recovered from the milk and urine of the nurse, but never from the child's urine.

5. *Mercury.*—The transmission of mercury from the nurse to the mother is very slight and inconstant.

6. The influence of the nurse's diet on the child is illusory; nurses can with impunity eat sour articles (lemons, vinegar,) without thereby influencing the child.

7. *Narcotics.*—(*a*) Tincture of opium in twenty to twenty-five drop doses. Thornhill claims to have observed a prolongation of the sleep in infants, while Fehling saw neither prolongation of sleep nor constipation resulting from it. (*b*) Hydrochlorate of morphine. The drug given in medicinal doses does not influence the child. (*c*) Chloral. Dose, fifteen to forty-five grains. Average length of sleep produced in nurse, two hours. No effects on the child are observable if it is strong and vigorous. If the child is weak and possibly born before the full term, it is advisable to wait two hours after administration of the drug to the nurse before allowing it to suckle. (*a*) Sulphate of atropine. Injected in the usual doses hypodermically in the nurse, the drug produces very distinct physiological effects in the child. The dilatation of the pupils taking place in the child does not disappear before twenty-four hours; hence, minute doses of the drug exclusively are permissible.—*Therapeutic Gazette.*

